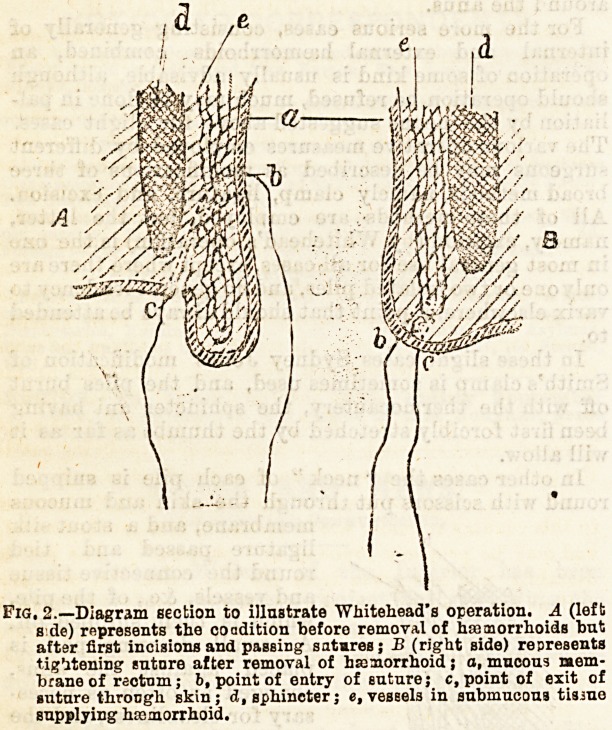# Hæmorrhoids

**Published:** 1893-01-07

**Authors:** 


### THE HOSPITAL CLINIC.

[*The Editor will be glad to receive offers of co-operation and contributions from members of the profession. All letters should be addressed to* THE EDITOR, THE LODGE, PORCHESTER SQUARE, LONDON, W.]

### ST. THOMAS'S HOSPITAL.

### HÆMORRHOIDS.

Among the many complaints to which the increasing proportion of sedentary to active occupations in modern civilisation is conducive, hæmorrhoids certainly take an important place, and the treatment of them in their various degrees forms a considerable element in the surgical practice of any "town" hospital, and particularly a London one. We have, therefore, considered that the following remarks, based upon notes of the practice of St. Thomas's Hospital, may prove of interest to the readers of this paper.

Following the usual plan of classification, we take first the treatment of the external hæmorrhoids, and we may note that in these cases it is not, as a rule, the hæmorrhoid as such that the patient complains of, but rather the discomfort or pain due to some associated condition, such as an anal fissure or eczema. The hæmorrhoid is the sign of a varicose condition of the rectal veins, and the consequent impaired vitality of the parts, with the irritation of the excessive mucous discharge from the congested mucous membrane, results first in a troublesome pruritus, which is then aggravated into an eczema, often with actual fissures, the latter sometimes caused by the scatching of the patient. An excessive sweating is frequently complained of also, especially between the folds of the nates and in the perinæum—a further result of the passive congestion, tending to keep up the eczema. In such cases it is often advisable to snip off the hæmorrhoid, usually forming little more than a loose "tab" of skin, just as one cauterises the protuberant granulations of a chronic ulcer; but the essential part of the treatment lies in allaying the irritation due to the eczema or associated fissures, for which various ointments are useful, a favourite one consisting of unguent: zinci with calomel (gr. x. ad ℥i.), and a small quantity of creolin, or liquor carbonis detergens (of the latter ♏ x. ad ℥i.) added. Where excessive local sweating is troublesome, a powder consisting of oxide of zinc gr. xx., starch gr. xx., and calomel gr. v., is useful for dusting over the parts affected. Fissures, if present, will frequently heal under the above treatment, but occasionally may require the application of nitric acid, or an incision into the sphincter to give them the necessary rest for healing.

At the same time general measures to prevent constipation and to restore the tone of the lower bowel should be attended to; strong purgatives should be avoided as a rule, but the combination of a small dose of belladonna and nux vomica (gr. $\frac{1}{4}$ to $\frac{1}{2}$ each), with cascara or aloin taken regularly for a time, and gradually discontinued by diminishing doses, will in time effect an improvement. Regular exercise and regular habits generally should also be advised.

It occasionally happens that a patient comes with an inflamed and thrombosed external hæmorrhoid. This condition is entremely painful, but can readily be cured by an incision through the centre of the "pile," by which the clot can be turned out. The relief is rapid, and no bleeding of any account occurs, the incision soon healing under the application of some ointment such as that mentioned above.

Before passing on to more serious conditions, we may remark that the popular idea of "piles" embraces all conditions of soreness or irritation about the anus, and it seems hardly too much to say that of all the cases of men under 30 applying at the out-patient department complaining of "piles," 80 or 90 per cent. will prove only to be suffering from mucous tubercles around the anus.

For the more serious cases, consisting generally of internal and external hæmorrhoids combined, an operation of some kind is usually advisable, although should operation be refused, much may be done in palliation by the means suggested above for slight cases. The various operative measures employed by different surgeons may be described as modifications of three broad methods, namely clamp, ligature, and excision. All of these methods are employed, but the latter, namely, excision (by Whitehead's operation) is the one in most general use for all cases, except where there are only one or two isolated piles, and no general tendency to varix elsewhere, a point that should always be attended to.

In these slight cases Sydney Jones' modification of Smith's clamp is sometimes used, and the piles burnt off with the thermocautery, the sphincter ani having been first forcibly stretched by the thumbs as far as it will allow.

In other cases the "neck" of each pile is snipped round with scissors put through the skin and mucous membrane, and a stout silk ligature passed and tied round the connective tissue and vessels, &c., of the pile, which is then snipped off. A small dressing pad is placed against the anus, changed as often as necessary for cleanliness, and the patient kept in bed for about ten days or a little more. The bowels may be opened by an aperient on the sixth day, before which little, if any, solid food should, as a rule, be given. Where a ligature has been used it will, as a rule, separate of itself in about a week.

FIG. 1.—Diagrammatic section of single hæmorrhoid, ligatured. The dotted line marks the point through which it is cut on removal, leaving the ligature to separate by sloughing. *a*, skin; *b*, sphincter; *c*, submucous tissue: *d*, mucous membrane of rectum.

Crushing the piles by the various forms of crushing clamps seems to have been very rarely practised.

In the more serious cases where the piles are large, or form a ring completely around the anus, Whitehead's operation, practically an "excision of the anus," is the method almost invariably selected.

The rectum having been carefully emptied by aperients and enema in the usual way, and the patient anæsthetised, the parts should be carefully scrubbed with soft soap and washed with some antiseptic solution, and the sphincter stretched as far as possible by the thumbs. The hæmorrhoids are then seized with a pair of clamp forceps at the point selected for beginning the incision. By snipping with blunt-ended scissors through the junction of skin and mucous membrane, or, in cases where many external piles are present, around the edge of the pile-bearing area, the piles can be drawn well down, and by a process of blunt dissection with the closed scissors separated from the parts below. A succession of fine silk sutures are then passed from within through the healthy mucous membrane just above the pile-bearing area and brought out through the skin at the margin of the incision.

The piles thus partly freed are now removed by snipping with scissors through the mucous membrane just below the point of entry of the sutures. Bleeding is free for the moment, but usually ceases when the sutures are drawn tight, and such vessels as may require it have been caught up with pressure-forceps,

FIG. 2.—Diagram section to illustrate Whitehead's operation. *A* (left side) represents the condition before removal of hæmorrhoids but after first incisions and passing sutures; *B* (right side) represents tightening suture after removal of hæmorrhoid; *a*, mucous membrane of rectum; *b*, point of entry of suture; *c*, point of exit of suture through skin; *d*, sphincter; *e*, vessels in submucous tissue supplying hæmorrhoid.

and, if necessary, ligatured. We may note that if the sutures are carefully passed they practically form ligatures to the vessels of the piles, and it is often remarkable, even in extensive cases with large vascular piles, how very few vessels need a separate ligature.

This process of snipping through the outer margin of the pile area, dissecting of the piles from the bowel wall, passing the sutures, snipping away the piles, and tying the sutures is repeated with section after section of the pile area till the circuit is complete, when if the suturing has been carefully performed there will be in place of the pile area a circular line of incision around the anus, but no exposed raw surface, for the healthy edge of mucous membrance should be accurately adjusted to the skin. With the exception of the trivial suppuration around the sutures, which allows them to come away, union by first intention may be expected. If the sphincter has been properly stretched beforehand there will be no pain to speak of, and in ten days or a fortnight the patient is usually able to get up. Retention of urine is generally present for the first day or two, as after all operations for piles, but provided there is no stricture—a point, of course, that should always be investigated before the operation—it is easily relieved by a soft Jacque's catheter.

The bowels may be opened by an aperient about the fourth or fifth day, before which the diet should be limited in the usual way, and occasional bathing with some antiseptic lotion will easily keep the parts clean. The sutures generally separate themselves without difficulty in about a week.

The experience of this hospital supports Whitehead's original contention that, so far from the operation leaving a stricture of the anus, as some have supposed would occur, there is less reason to fear this than after extensive ligature operations, for no raw surface is left to granulate and cicatrise.

In severe cases, where actual strangulation and sloughing has occurred before the patient has sought advice, it is generally best to clean up the parts by a few days of poulticing and bathing, during which a "spontaneous cure" is partly effected by the separation of the sloughs. Such cases may be left without operation, but as a rule it is considered best to do a small operation on the lines of Whitehead's operation as soon as the parts have quieted down sufficiently to allow it.

## Figures and Tables

**Fig. 1. f1:**
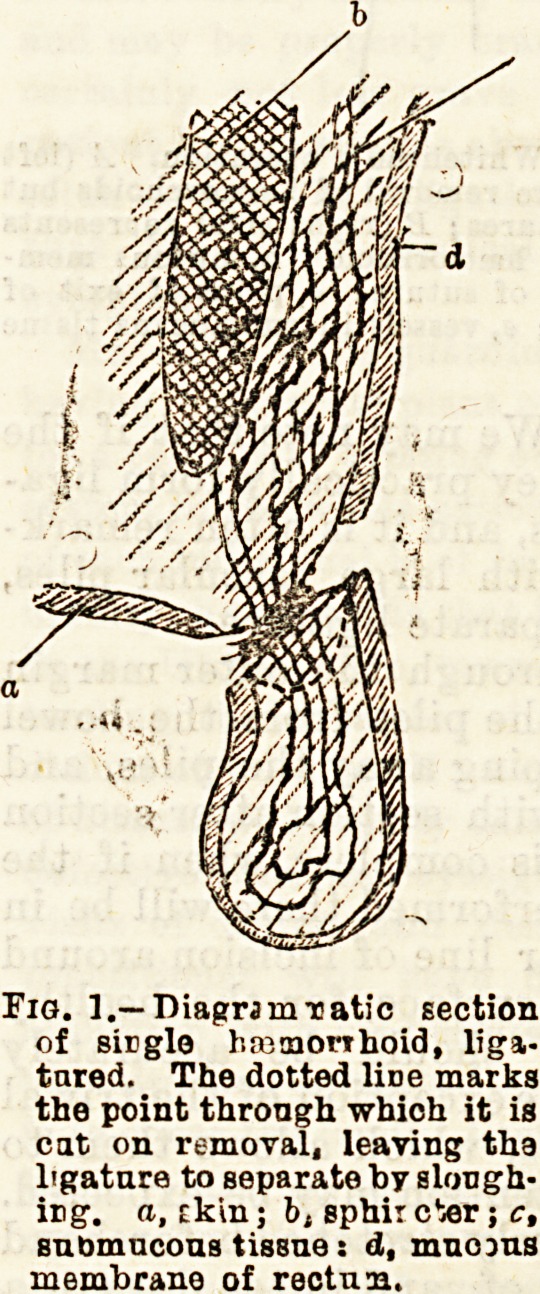


**Fig. 2. f2:**